# Mapping macrophage polarization over the myocardial infarction time continuum

**DOI:** 10.1007/s00395-018-0686-x

**Published:** 2018-06-04

**Authors:** Alan J. Mouton, Kristine Y. DeLeon-Pennell, Osvaldo J. Rivera Gonzalez, Elizabeth R. Flynn, Tom C. Freeman, Jeffrey J. Saucerman, Michael R. Garrett, Yonggang Ma, Romain Harmancey, Merry L. Lindsey

**Affiliations:** 1 Department of Physiology and Biophysics, Mississippi Center for Heart Research, University of Mississippi Medical Center, 2500 North State St., Jackson, MS 39216-4505 USA; 2 Research Service, G.V. (Sonny) Montgomery Veterans Affairs Medical Center, Jackson, MS 39216 USA; 3 The Roslin Institute, University of Edinburgh, Easter Bush, Midlothian, Scotland UK; 4 Department of Biomedical Engineering, University of Virginia, Charlottesville, VA USA; 5 Department of Pharmacology and Toxicology, University of Mississippi Medical Center, Jackson, MS 39216 USA

**Keywords:** Myocardial infarction, Macrophage, Transcriptome, RNA-Seq, LV remodeling

## Abstract

In response to myocardial infarction (MI), cardiac macrophages regulate inflammation and scar formation. We hypothesized that macrophages undergo polarization state changes over the MI time course and assessed macrophage polarization transcriptomic signatures over the first week of MI. C57BL/6 J male mice (3–6 months old) were subjected to permanent coronary artery ligation to induce MI, and macrophages were isolated from the infarct region at days 1, 3, and 7 post-MI. Day 0, no MI resident cardiac macrophages served as the negative MI control. Whole transcriptome analysis was performed using RNA-sequencing on *n* = 4 pooled sets for each time. Day 1 macrophages displayed a unique pro-inflammatory, extracellular matrix (ECM)-degrading signature. By flow cytometry, day 0 macrophages were largely F4/80^high^Ly6C^low^ resident macrophages, whereas day 1 macrophages were largely F4/80^low^Ly6C^high^ infiltrating monocytes. Day 3 macrophages exhibited increased proliferation and phagocytosis, and expression of genes related to mitochondrial function and oxidative phosphorylation, indicative of metabolic reprogramming. Day 7 macrophages displayed a pro-reparative signature enriched for genes involved in ECM remodeling and scar formation. By triple in situ hybridization, day 7 infarct macrophages in vivo expressed collagen I and periostin mRNA. Our results indicate macrophages show distinct gene expression profiles over the first week of MI, with metabolic reprogramming important for polarization. In addition to serving as indirect mediators of ECM remodeling, macrophages are a direct source of ECM components. Our study is the first to report the detailed changes in the macrophage transcriptome over the first week of MI.

**Electronic supplementary material:**

The online version of this article (10.1007/s00395-018-0686-x) contains supplementary material, which is available to authorized users.

## Introduction

Myocardial infarction (MI) invokes a cardiac wound healing response that involves early initiation of inflammation, followed by robust scar formation in the infarct area. The macrophage is a key regulator of cardiac remodeling, providing both strong pro-inflammatory signals early and reparative cues later [13, 24, 25, 41, 44]. Macrophages naturally reside in the healthy heart, largely derived from the embryonic yolk sac, proliferating locally and overseeing normal tissue maintenance [22, 34].

In response to MI, circulating pro-inflammatory Ly6C^high^ monocytes are rapidly recruited from bone marrow and splenic reservoirs to the infarcted area by the CC chemokine CCL2/MCP-1, and require CCR2 (the endogenous receptor for CCL2) for extravasation [14, 80]. Early post-MI, monocyte-derived macrophages promote inflammation through release of pro-inflammatory cytokines such as interleukin (IL)-1β, a key regulator of post-MI inflammation [70].

In the healthy heart and following MI, monocyte/macrophage subpopulations are identified by a limited number of cell surface markers. Resident cardiac macrophages express high levels of the myeloid marker CD11b, as well as canonical macrophage markers including CD14, CD86, CX3CR1, F4/80, and MHC-II, and display an anti-inflammatory M2 phenotype [63]. Resident macrophages are characterized by high expression of F4/80, while infiltrating monocytes and monocyte-derived macrophages express F4/80 at lower levels [22, 62]. Following MI, F4/80^high^Ly6C^low^ resident macrophages are rapidly replaced by pro-inflammatory infiltrating F4/80^low^Ly6C^high^ monocytes that are CCR2^high^ [22]; by post-MI day 7, the macrophage phenotype shifts to a predominantly anti-inflammatory/pro-reparative M2 phenotype.

Specifically targeting macrophages to improve MI outcomes has proven both promising and challenging, as therapeutic approaches successfully manipulating the macrophage depend on both temporal and spatial factors [13, 18]. For example, the early inflammatory response is critical for wound healing, as too little or excess inflammation can adversely affect remodeling; the same paradigm is true for the later reparative and fibrotic response [18]. While macrophages have been extensively studied in steady state and aging hearts [10, 49, 61], as well as following pressure overload [88], the full picture of the macrophage evolution over the first week after MI has not been developed.

Accordingly, the objective of this study was to combine transcriptomics, flow cytometry, and cell physiology to provide a map of macrophage phenotypes in response to MI. We analyzed transcriptomic changes at days 1, 3, and 7 post-MI to reflect the early inflammatory, proliferative, and maturation phases. We hypothesized that macrophages would undergo changes over the MI time course that range from pro-inflammatory to reparative polarization. To our knowledge, this is the first study to report in detail the full transcriptome changes that occur in cardiac macrophages that mediate post-MI wound healing and remodeling.

## Methods

### Animal use

All procedures involving animals were approved by the Institutional Animal Care and Use Committee at the University of Mississippi Medical Center. A total of 122 C57BL/6J adult (3–6 month old) male mice were used for this study. Mice within this age range are at similar physiological maturation [26]. Groups were randomly assigned prior to the surgeries by one investigator (KYDP) and another investigator (YM) performed the majority of surgeries.

### Coronary artery ligation

To produce permanent MI, mice underwent coronary artery ligation surgery as described previously and according to the Guidelines for Experimental Models of Ischemia and Infarction [12, 28, 39, 47, 94]. Mice were anesthetized with 2% isoflurane, intubated, and ventilated. The left coronary artery was ligated with 8–0 suture, and MI was confirmed by left ventricle (LV) blanching and ST-segment elevation on the EKG. Mice were administered buprenorphine (0.05 mg/kg body weight) immediately before surgery.

### Echocardiography and necropsy

LV physiology was determined by transthoracic echocardiography (Vevo 2100, VisualSonics; Toronto, CA) as described before and according to the Guidelines for Measuring Cardiac Physiology in Mice [12, 28, 40, 47]. Mice were anesthetized under 1–2% isoflurane, and both long and short-axis images were obtained. Measurements were taken on the terminal day and were averaged from three cardiac cycles for each mouse. Following imaging, the hearts were removed and the left ventricle (LV) divided into remote and infarct (which included border zone) regions. Each region was separately weighed for infarct area estimation. The infarct sizes over the 3 MI time points had a coefficient of variation of 18%, indicating gene variation was not likely due to differences in infarct sizes.

### Isolation of LV infarct macrophages

LV macrophages were isolated from the infarct region by immunomagnetic separation as described previously [12, 28]. Excised LV tissue was rinsed and immediately minced and digested by collagenase II (Worthington; Lakewood, NJ) and DNase solution in Hanks buffered saline solution. After digestion, a single-cell suspension was generated and filtered through a 30 µm pre-separation column. Cell suspensions were incubated at 4 °C with an anti-Ly6G-biotin antibody (Miltenyi Biotech, Bergisch Gladbach, Germany, 130-092-332) to remove neutrophils, followed by an anti-CD11b-biotin antibody (Miltenyi 130-049-601) for 15 min, followed by anti-biotin microbeads (Miltenyi 130-092-332) for 10 min. Cells conjugated to the antibody microbeads were separated by magnetic columns (Miltenyi 130-042-201).

The average numbers of isolated macrophages ± SEM and the coefficient of variation (CV) from each of the individual pooled animals for each time point were as follows: day 0—1.62 × 10^5^ ± 0.18 × 10^5^, CV = 31%; day 1—2.61 × 10^5^ ± 0.33 × 10^5^, CV = 43%; day 3—1.75 × 10^6^ ± 0.08 × 10^6^, CV = 34%; and day 7—7.64 × 10^5^ ± 0.46 × 10^5^, CV = 17%. To evaluate whether pooling increased variability, we compared the CV of the day 7 MI macrophage pools with the CV of a previously published day 7 MI macrophage RNA-seq experiment that used macrophages isolated from individual mice [28]. The CV for the day 7 pooled set was 17%, while the CV for the individual mice was 48% (*n* = 5). There was no indication, therefore, that pooling increased variability.

An initial assessment of macrophages freshly isolated from the myocardium yielded RNA that was not of sufficient quality for RNA-sequencing (RNA-Seq). To determine an optimal culturing time, day 3 post-MI CD11b^+^Ly6G^−^ macrophages (1.5 × 10^6^ cells/well) were plated in 6-well culture dishes for 2 or 20 h in RPMI 1640 medium supplemented with 0.1% FBS and 1% antibiotics. After incubation, non-adherent cells were washed off and the remaining adherent cells were used for transcriptomics analysis by RNA-Seq. The 20 h culturing changed macrophage phenotype; Supplemental Fig. 1), and based on this result, 2 h was selected as the incubation period for the time course evaluation.

### RNA-Seq

To obtain high-quality RNA for sequencing (from day 0 hearts in particular), macrophages were pooled from *n* = 81 hearts to obtain four biological replicates of 1.5 × 10^6^ cells for each day post-MI. Whole transcriptome analysis was performed as described previously [28, 47]. RNA was extracted using the Pure Link RNA Mini Kit (Ambion, Foster City, CA) according to manufacturer instructions and assessed for quality control parameters of minimum concentration and size range. cDNA libraries were developed using the TruSeq Total Stranded RNA with RiboZero Kit (Ambion), set-A, quantified with the Qubit System (Invitrogen, Carlsbad, CA), and assessed for quality and size with the Experion DNA 1K Chip (Bio-Rad, Hercules, CA). The libraries (*n* = 12 pooled samples per library) were sequenced using the NextSeq 500 High Output Kit (300 cycles, paired end 100 bp) on the Illumina NextSeq 500 platform (Illumina, San Diego, CA). Sequenced reads (*n* = 30–50; Cloud Computing Platform), and Fastq sequence files were used to align reads to the reference genome USCS-GRCm38/mm10) using RNA-Seq Alignment Application with STAR aligner. Fragments per kilobase of transcript per million mapped reads (FPKM) values of reference genes and transcripts were generated using Cufflinks 2. Variability (coefficient of variation) of pooled sets was compared to variability of a previous RNA-Seq experiment on day 7 post-MI macrophages from individual mice [28].

### Bioinformatic analyses

Analyses tools available in the online resource Metaboanalyst 3.0 (http://www.metaboanalyst.ca/) and GraphPad Prism were used for graphical and statistical analyses [92, 93]. FPKM values were uploaded into Metaboanalyst, and one-way ANOVA with Tukey’s post hoc test was performed to determine differentially expressed genes (defined as false discovery rate (FDR) adjusted *p* < 0.05). For individual post-MI days, differential expression was characterized by a fold change threshold of > 2.0 or < 0.5 compared to day 0 no MI values and a *p* value of < 0.05 by unpaired two-tailed *t* test. Markov clustering analysis was performed independently by two investigators (AJM and TCF) using Graphia Pro software (Kajeka, Edinburgh, UK) using genes with a pairwise Pearson correlation threshold of *r* > 0.95. Both investigators obtained similar results. Enrichment analysis for differentially expressed genes was performed using Enrichr (http://amp.pharm.msm.edu/Enrichr/) gene ontology (GO) biological processes and Ingenuity Pathway Analysis (Qiagen) canonical pathways. For GO terms, the combined score (calculated from *Z*-score and *p* value) was reported.

### RT-PCR validation

A total of five genes (Arg1, Ifng, Il1b, Lgals3, and Tnf) were evaluated by quantitative RT-PCR on the same macrophage RNA samples used for RNA-Seq and assessed for correlation. RNA was reverse transcribed to cDNA using the High Capacity RNA-to-cDNA kit (Applied Biosystems 4387406). Gene expression was quantified using the Taqman Gene Expression Assay and primers for Arg1, Ifng, Il1b, Lgals3, and Tnf (Applied Biosystems). Values for the arrays were normalized to the housekeeping gene Hprt1.

### Flow cytometry

LV tissue excised from day 0 and day 1 post-MI mice was minced and digested with 600 U/ml collagenase II (Worthington, LS004177, Lot 47E17554B) and 60 U/ml DNase I in Hanks buffered saline solution and filtered through a 30-µm separation filter to generate single-cell suspensions. Red blood cells were lysed (Red Blood Cell Lysis Solution, Miltenyi 130-094-183) and non-specific interactions were blocked with FcR Blocking Reagent (Miltenyi 130-092-575). Cells were stained with the following fluorophore-conjugated antibody panels: CD45-FITC (Miltenyi 130-110-658), CD11b-APC-Vio770^®^ (Miltenyi 130-109-288), F4/80-PerCP-Vio700 (Miltenyi 130-102-161), Ly6C-VioBlue^®^ (Miltenyi 130-111-921), and Ly6G-APC (Miltenyi 130-107-914). Samples were quantified using the MACSQuant Analyzer 10 (Miltenyi). Cell populations were gated on live singlets, with cells from monocyte-derived/macrophage lineage classified as CD45^+^CD11b^+^Ly6G^−^ cells.

### In vivo phagocytosis assay

To evaluate macrophage phagocytosis, day 0 or day 3 post-MI mice were injected with 100 µg of fluorescein-labeled Escherichia coli K-12 BioParticles (Molecular Probes, Eugene, OR, V-6694) through the jugular vein [22]. After 2 h, cardiac macrophages were isolated, cultured for 2 h to remove unattached cells, and fixed with 100% ethanol. Nuclei were stained with DAPI. Images were acquired using an Olympus IX81 microscope. Phagocytic macrophages (green fluorescence) were counted as a percentage of the total cells per field.

### In vivo proliferation assay

To evaluate macrophage proliferation, day 0 or day 3 post-MI mice were injected with 1 mg BrdU (Sigma, St. Louis, MO, 11647229001) intraperitoneally 2 h before being killed [22]. Isolated infarct macrophages were adhered to slides, fixed with 100% ethanol, permeabilized with Triton-X 100, and stained with anti-BrdU-FITC antibody (eBioscience, Waltham, MA, 11-5071-42, 1:20). Nuclei were stained with DAPI. Images were acquired using an Olympus IX81 microscope. Proliferating cells (green fluorescence) were counted as a percentage of total cells per field.

### In vivo macrophage turnover

To evaluate macrophage turnover, mice were injected with a FITC-F4/80 antibody (Biolegend, San Diego, CA, 123107, 200 µg/kg) through the jugular vein at 24 h post-MI (day 1) and killed at day 3 post-MI. MI mice without injection served as negative controls. Infarct macrophages were isolated, and FITC + cells were quantified using a MACSQuant Analyzer 10. The data were analyzed using the MACSQuantify software and were presented as the percentage of FITC^+^ cells to total macrophages.

### Triple in situ hybridization

Day 7 post-MI LV sections (*n* = 3) collected from the mid-papillary region were fixed in 10% zinc-buffered formalin for 24 h at room temperature paraffin-embedded, and sectioned at 5 µm. In situ hybridization was performed with three probe sets per section using the RNAscope Multiplex Fluorescent Reagent Kit v2 (Advanced Cell Diagnostics, Newark, CA). Samples were hybridized using probes (all from Advanced Cell Diagnostics) specific for Acta2 (319531, 1:1000), Ccr2 (433271, 1:1000), Col1a1 (319371, 1:1500), Emr1 (317961, 1:1000), and Postn (418581, 1:1500); nuclei were stained with 4′,6-diamidino-2-phenylindole (DAPI). Probes were conjugated to the following fluorophores (Perkin Elmer, Waltham, MA, TSA Plus): fluorescein (NEL741E001KT), Cy3 (NEL744E001KT), or Cy5 (NEL745E001KT). Images were acquired at 40× using the Mantra Quantitative Pathology Imaging System (Perkin Elmer), and analyses were performed using the cell phenotyping feature in the inForm software (Perkin Elmer).

### Statistics

All experiments were performed and analyzed in a blinded design. Data are presented as mean ± SEM. Survival rate was analyzed by Kaplan–Meier survival analysis and compared by the log rank test. For echocardiography, comparisons were made using one-way ANOVA followed by Tukey’s post hoc test. Statistics and bioinformatics for the RNA-sequencing are described above. RNA-sequencing comparisons to quantitative RT-PCR were made by Pearson’s linear regression analysis. Two group comparisons were analyzed by unpaired two-tailed *t* test. A value of *p* < 0.05 was considered statistically significant.

## Results

### Macrophages continually polarize over the post-MI time continuum

#### Proof of successful MI

Day 7 post-MI survival was 56% (Supplemental Fig. 2a), consistent with others and our own past reports [11, 28, 43, 75, 87, 96]. Of the day 7 mice that did not survive, 58% (7/12) died from cardiac rupture, as assessed at autopsy. Infarct areas (% LVI mass by total LV mass) were similar among days 1, 3, and 7 MI groups (Supplemental Fig. 2b). As expected, cardiac physiology was impaired after MI, with LV infarct wall thinning and dilatation evident beginning at day 1 after MI (Supplemental Fig. 2c–e). Fractional shortening decreased similarly across all post-MI groups (Supplemental Fig. 2f).

#### Proof of cell isolation purity

Supplemental Fig. 3 shows FPKM values plotted for macrophage specific markers (Cd14, Cd68, Emr1, Fcgr3, Itgam, and Lgals3) and compared to non-macrophage cell-specific markers for endothelial, fibroblast, lymphocyte, myocyte, and neutrophil cell types. FPKM values for macrophage specific markers averaged 455, while for non-macrophage specific markers, the average FPKM was ~ 3. These results indicate that our macrophage isolations were ultra-pure and free of contamination from other cell types.

#### Differentially expressed genes

**Fig. 1:**
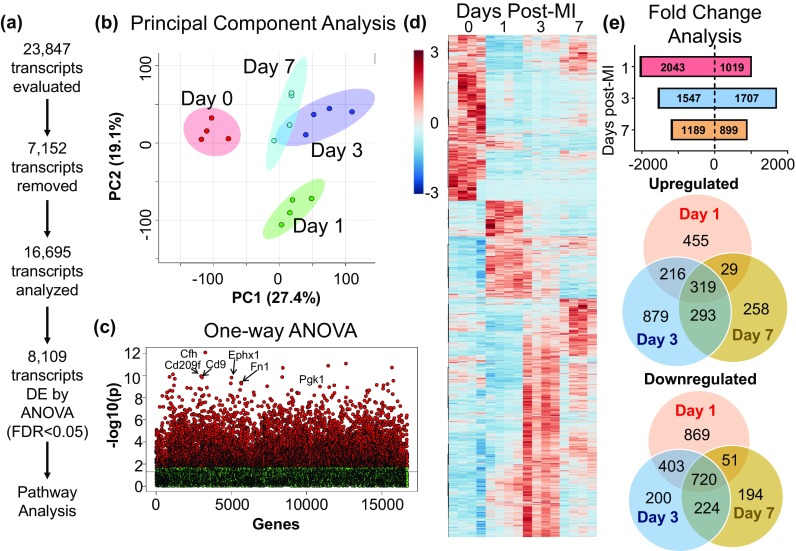
Distinct time-dependent gene expression profiles in post-MI macrophages. **a** Of 23,847 genes sequenced, 7152 did not meet quality control standards and were removed. Of the remaining 16,695 genes, 8109 were differentially expressed (DE) by one-way ANOVA (FDR adjusted *p* value < 0.05). **b** Macrophages from different post-MI days analyzed by principal component analysis. Day 1 macrophages were most distinct from the other times. Day 3 and 7 macrophages were distinct from day 0 and day 1 and showed overlap. **c** One-way ANOVA plot showing significant genes in red and **d** heat map of all differentially expressed genes. **e** Fold change analysis of differentially expressed genes at each day post-MI (fold change threshold of 2, FDR adjusted *p* value < 0.05) and Venn diagrams of upregulated and downregulated genes showing distinction and overlap in gene expression among the times

From the 23,847 genes in the dataset, 4064 were removed from analysis as FPKM values were 0 for all biological replicates in all four groups and 2938 were removed as there was < 3 replicates with values > 0 for any one group. Of the remaining 16,845 genes, 150 had duplicate or triplicate measurements that were removed. This left a total of 16,695 unique transcripts for analysis (Fig. 1a; Supplemental Table 1). By principal component analysis, each of the days separated out into its own pattern, indicating that each day had a unique gene expression profile (Fig. 1b). By one-way ANOVA with Tukey’s post hoc test, 8109 genes differed among groups (with FDR adjusted *p* < 0.05; Fig. 1c, heat map Fig. 1d). By fold change analysis using cut-off points of two for fold change and *p* < 0.05 for *p* value by unpaired two-tailed *t* test, day 1 had 6% of genes (1019) significantly upregulated and 12% (2043) downregulated; day 3 had 10% (1707) upregulated and 9% (1547) downregulated, and day 7 had 5% (899) upregulated and 7% (1189) downregulated compared to day 0 macrophages (Fig. 1e). Fold change analysis of commonly used M1 and M2 markers is displayed in Supplemental Fig. 4.

RT-PCR validation of FPKM values was performed for Il1b, Arg1, Lgals3, Tnf, and Ifng on the same samples used for RNA-Seq (Supplemental Fig. 5). These genes were chosen based on their strong association with macrophages. Positive correlations for Il1b (*r* = 0.99, *p* < 0.0001), Arg1 (*r *= 0.95, *p* < 0.0001), and Lgals3 (*r* = 0.51, *p* = 0.02) were observed. Tnf and Ifng showed low agreement due to low detection by RT-PCR, either due to sensitivity issues or primer design.

#### Pattern clustering

**Fig. 2:**
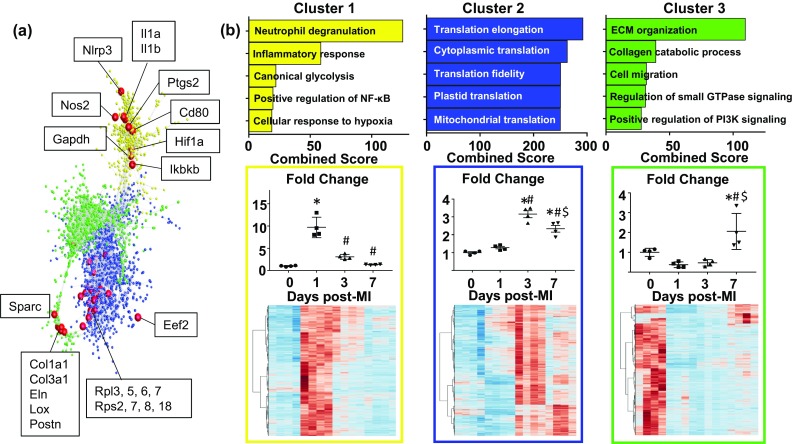
Gene expression pattern clustering. **a** Markov clustering analysis generated three distinct major clusters representing different post-MI gene expression patterns. Each node represents a single gene, and genes within the same cluster (color) show similar gene expression patterns. Genes representing each cluster are highlighted in red. **b** Enrichment analysis and expression patterns for each cluster. Cluster 1 (yellow) contained 565 genes increased at day 1 and was enriched for pro-inflammatory processes. Cluster 2 genes (blue, 1965 genes) were increased at day 3 and 7 and were enriched for translation processes. Cluster 3 (green, 1222 genes) were increased at day 7 and enriched for ECM processes. *p < 0.05 versus day 0, ^#^*p* < 0.05 versus day 1, ^$^*p* < 0.05 versus day 3

Gene expression patterns across the post-MI remodeling spectrum were also analyzed by Markov pattern clustering. A total of three major clusters distinguished the time points (Fig. 2a), with representative genes from each cluster highlighted. Cluster 1 (yellow, 565 genes) showed an overall expression pattern of mRNAs being increased only at day 1 after MI compared to day 0 (Fig. 2b). GO enrichment for this cluster revealed that pro-inflammation (neutrophil degranulation, *p* = 3.2E−11; inflammatory response, *p* = 7.5E−7; canonical glycolysis, *p* = 5.3E−7; positive regulation of NF-κB activity, *p* = 0.0018; cellular response to hypoxia, *p* = 0.00082) was the major process represented by these genes, suggesting that day 1 macrophages rapidly upregulate inflammatory and glycolytic processes which are then rapidly turned off. The neutrophil degranulation term is term overlap and does not imply neutrophil contamination, as we saw essentially no expression of neutrophil-specific markers, and genes in our analysis represented by this term are known to be expressed in macrophages (e.g., Mmp8/9, Mif, and Lgals3). Cluster 2 (blue, 1965 genes) showed a pattern of increasing at day 3 and day 7, with a peak at day 3. GO processes related to mRNA translation were prominent in this cluster. Cluster 3 (green, 1222 genes) showed a pattern of being decreased at days 1 and 3, and either increased or decreased at day 7. The top two GO processes were extracellular matrix (ECM) organization (*p* = 1.06E−16) and collagen catabolic process (*p* = 3.3E−7), reflecting direct upregulation of some, but not all ECM genes and indirect regulation of ECM processes.

### Day 1 MI macrophages display a pro-inflammatory monocyte-derived signature

**Fig. 3:**
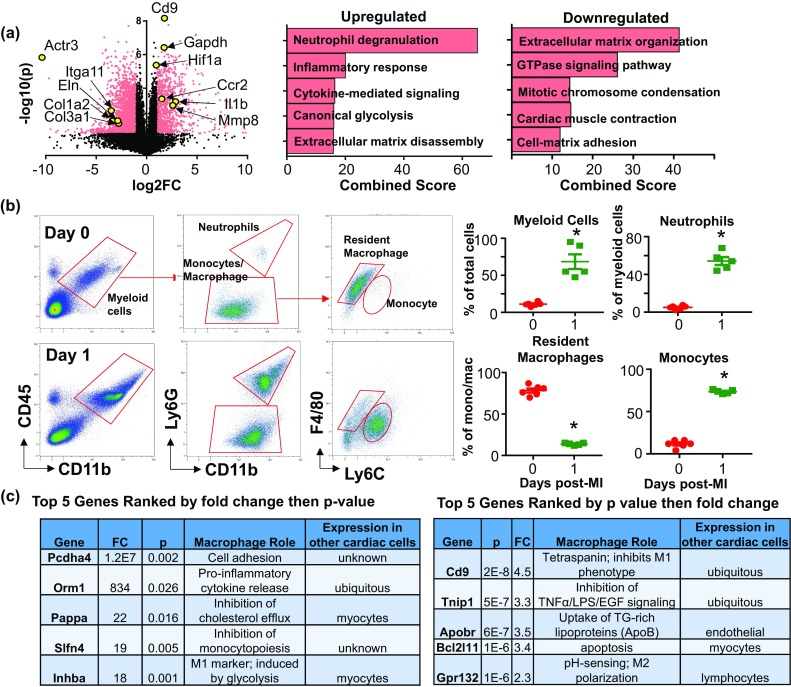
Day 1 post-MI macrophages showed a pro-inflammatory profile. **a** Volcano plot with all values normalized to day 0 no MI controls and representative genes highlighted (left). Enrichment analysis of upregulated and downregulated genes (right). **b** Flow cytometry analysis of macrophage cell surface markers. At day 1 post-MI, total myeloid cells, neutrophils, and monocytes significantly increased in the infarct region, while resident macrophages decreased. **c** Top five upregulated day 1 post-MI genes ranked by fold change (left) and *p* value (right). *p < 0.05 versus day 0

Genes differentially expressed at day 1 post-MI are displayed by volcano plot (Fig. 3a), with representative genes from each GO process highlighted. The representative genes were the top five ranked up- and down-regulated genes based on fold change (≥ 2) and *p* value at each time point. Notable upregulated GO processes included inflammatory response (*p* = 0.007), cytokine-mediated signaling (*p* = 0.002), canonical glycolysis (*p* = 0.002), ECM disassembly (*p* = 0.002), and cellular response to hypoxia (*p* = 0.02; Fig. 3a). Downregulated biological processes at day 1 included ECM organization (*p* = 9.3E−7) and cell–matrix adhesion (*p* = 0.007).

To determine macrophage subpopulation heterogeneity within the 1 day post-MI time, we performed multi-marker flow cytometry. Myeloid cells (neutrophils and macrophages) were elevated (27 ± 4% of total cells vs. 4 ± 1% at day 0, a 6.2-fold increase; Fig. 3b). We further gated on the myeloid cell population to differentiate monocytes and macrophages from neutrophils based on Ly6G expression. Ly6G + neutrophils were significantly increased in the infarct region (54 ± 4% of the total myeloid cells, vs. 5 ± 1% at day 0, 10.8-fold increase). Gating on CD45 + CD11b + Ly6G- cells, F4/80 and Ly6C further divided this population into resident macrophages (F4/80^high^Ly6C^low^) and infiltrating monocytes (F4/80^low^Ly6C^high^). Resident macrophages were significantly decreased in the infarct (14 ± 1% of at day 1 vs. 78 ± 3% at day 0, 5.7-fold decrease) while monocytes were significantly increased (74 ± 1% at day 1 vs. 12 ± 2% at day 0). These results are consistent with what others have shown indicating that infiltrating monocytes replace resident macrophages early after MI [13, 14, 22, 54, 62, 63].

**Fig. 4:**
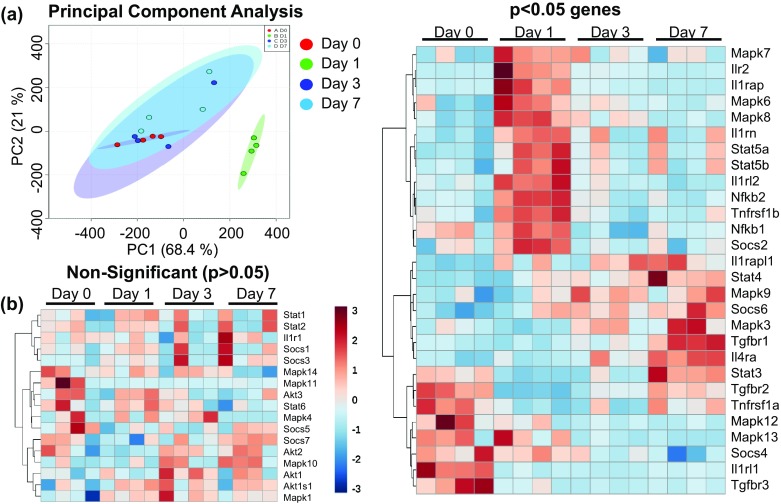
Post-MI macrophage signaling profiles. **a** Principal component analysis of genes involved in ubiquitous signaling pathways indicates that day 1 macrophages display a unique signaling profile compared to days 0, 3, and 7. **b** Heat maps grouped by significance (left, non-significant genes (*p* > 0.05); right, significant genes (*p* < 0.05) by unpaired two-tailed *t* test)

To define the day 1 post-MI macrophage, we ranked the top five uniquely upregulated genes by fold change followed by *p* value, and the top five ranked by *p* value followed by fold change (Fig. 3c). All of top ten ranked genes had previously been associated with macrophages: Pcdha4 [15], Orm1 [38], Pappa [78], Slfn4 [82], Inhba [37], Cd9 [74], Tnip1 [95], Apobr [79], Bcl2l11 (Bim) [30], and Gpr132 [4]. Day 1 macrophages displayed a unique signaling profile by principal component analysis compared to the other three time points (Fig. 4a), with expression of genes associated with IL-1, TNF, NF-κB, MAPK, STAT5, and SOCS2 signaling pathways (Fig. 4b).

### Day 3 MI macrophages are phagocytic, proliferative, and display a metabolic reprogramming signature

**Fig. 5:**
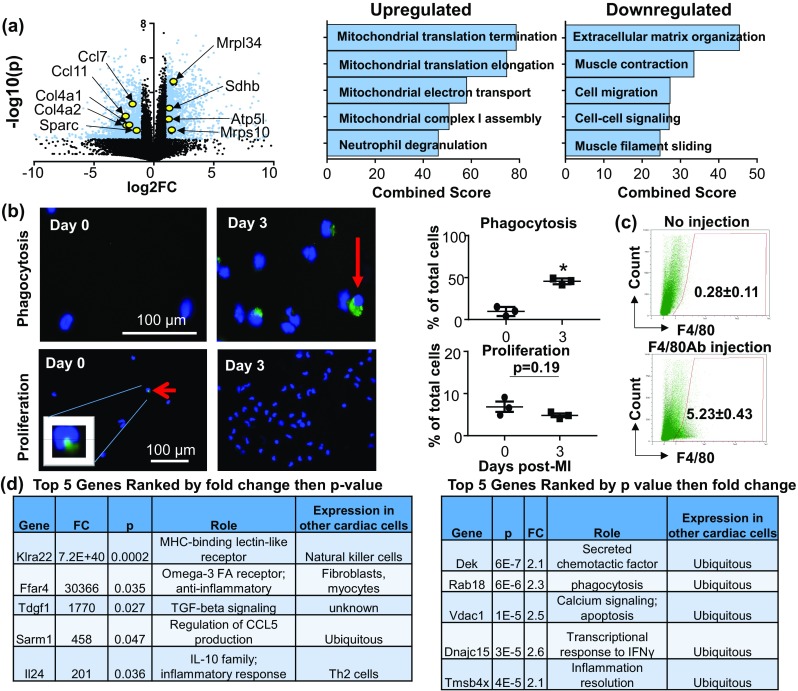
Day 3 post-MI macrophages showed a phagocytic, proliferative, and metabolic reprogramming profile. **a** Volcano plot with representative genes highlighted and enrichment analysis of upregulated and downregulated genes. All values are normalized to day 0 no MI controls and representative genes are highlighted. **b** Representative images of phagocytic (top) and proliferative (bottom) macrophages. Phagocytic capacity significantly increased at day 3 post-MI, whereas proliferation was similar to day 0. **c** In vivo turnover. At day 3 post-MI, ~ 5% of macrophages were remaining from day 1. **d** Top five upregulated day1 genes ranked by fold change (left) and *p* value (right). *p < 0.05 versus day 0

Genes differentially expressed at day 3 are displayed by volcano plot, with representative genes highlighted in yellow (Fig. 5a). The major upregulated GO processes were related to mitochondrial function, including mitochondrial translation termination (*p* = 8.4E−10) and elongation (*p* = 8.4E−10), electron transport (*p* = 4.4E−11), respiratory chain complex I assembly (*p* = 8.4E−10), cristae formation (*p* = 2.9E−5), and mitochondrial ATP synthesis-coupled proton transport (*p* = 7.1E−5). Downregulated GO processes included ECM organization (*p* = 2.4E−7), and cell migration (*p* = 0.0002). By IPA analysis, oxidative phosphorylation (42/109 genes; *p* = 2.5E−21) and mitochondrial dysfunction (51/171 genes; *p* = 8.0E−20) were the top upregulated canonical pathways, while leukocyte extravasation (38/211 genes; *p* = 2.9E−9) and agranulocyte adhesion and diapedesis (35/191 genes; *p* = 7.7E−9) were the major downregulated pathways (Supplemental Fig. 6).

We assessed in vivo macrophage phagocytosis, proliferation, and turnover. Macrophages from post-MI day 3 showed significantly increased phagocytosis compared to day 0 (Fig. 5b). In line with increased phagocytic capacity, IPA analysis indicated that phagosome maturation was an upregulated pathway at day 3 (20/148 genes; *p* = 0.003). Likewise, the phagosome maturation GO pathway was increased at day 7 post-MI (11/148 genes; *p* = 0.02), albeit to a lesser extent than day 3. Phagosome formation, but not maturation, was an upregulated pathway at day 1 (15/130 genes; *p* = 0.0003). While day 3 proliferation was not significantly different from day 0 (Fig. 5b), resident (day 0) macrophages have been shown to be proliferative [22]. Further, GO processes such as DNA replication initiation (*p* = 0.002), DNA replication (*p* = 0.01), and mitotic cell cycle (*p* = 0.01) were upregulated, indicating a proliferative signature at day 3. Genes associated with phagocytosis and proliferation are displayed in Supplemental Fig. 7. Macrophage turnover was assessed by F4/80 antibody injection at day 1 post-MI; only 5.2 ± 0.4% of macrophages at day 3 post-MI stained positive for the F4/80 antibody (Fig. 5c), indicating rapid turnover as reported by others [36].

To define the day 3 post-MI macrophage, we ranked the top five uniquely upregulated genes by fold change followed by *p* value, and the top five ranked by *p* value followed by fold change (Fig. 5d). All of the top ten ranked genes had previously been associated with macrophages: Klra22 [59], Ffar4 [91], Tdgf1 [72], Sarm1 [19], Il24 [73], Dek [50], Rab18 [21], Vdac1 [7], Dnajc15 [55], and Tmsb4x [83].

### Day 7 MI macrophages display a pro-reparative signature

**Fig. 6:**
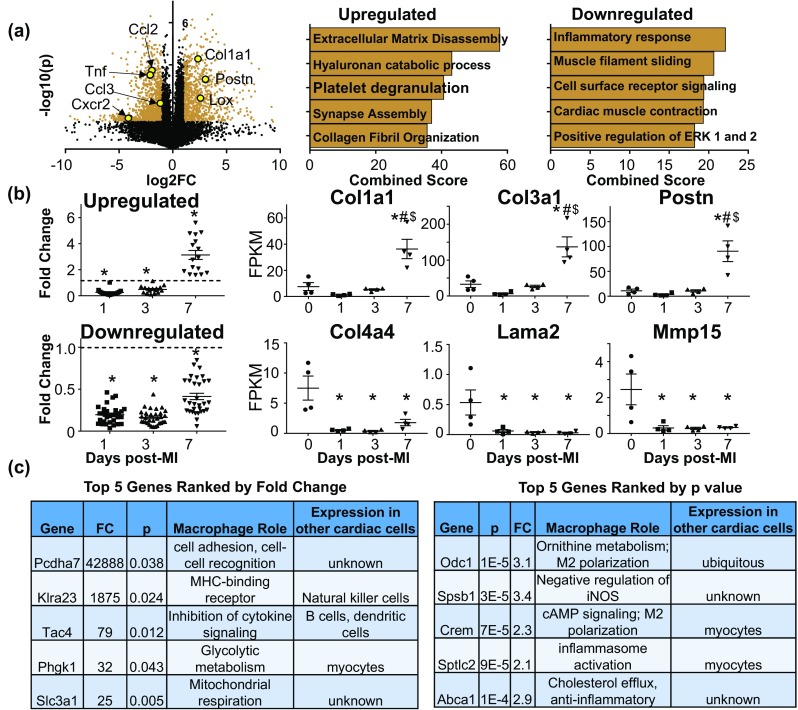
Day 7 post-MI macrophages showed a pro-reparative profile. **a** Volcano plot with representative genes highlighted and enrichment analysis of upregulated and downregulated genes. All values are normalized to day 0 no MI controls and representative genes are highlighted. **b** ECM genes that clustered together. Fold change values for genes upregulated at day 7 compared to day 1 and 3 post-MI (top) or remained downregulated at day 7 (bottom). **c** Top five upregulated day 7 genes ranked by fold change (left) and *p* value (right). *p < 0.05 versus day 0, ^#^*p* < 0.05 versus day 1, ^$^*p* < 0.05 versus day 3

Genes differentially expressed at day 7 post-MI are displayed by volcano plot, with representative genes highlighted in yellow (Fig. 6a). The major upregulated GO processes were related to ECM remodeling (Supplemental Table 2), including ECM disassembly (*p* = 0.0004) and collagen fibril organization (*p* = 0.01), and inflammatory response (*p* = 0.004) was the major downregulated GO process. By IPA analysis, inhibition of matrix metalloproteinases (MMPs) was a major upregulated canonical pathway (7/39 genes, *p* = 0.0004), while agranulocyte adhesion and diapedesis was a major downregulated pathway (34/192 genes, *p* = 4.6E−12; Supplemental Fig. 6). Clustering analysis identified a number of ECM genes downregulated at days 1 and 3 and either upregulated or downregulated at day 7 compared to day 0 (Fig. 6b; Supplemental Table 2).

To define the day 7 post-MI macrophage, we ranked the top five uniquely upregulated genes by fold change followed by *p* value, and the top five ranked by *p* value followed by fold change (Fig. 6c). All of the top ten ranked genes had previously been associated with macrophages: Pcdha7 [15], Klra23 [59], Tac4 [3], Phgk1 [16], Slc3a1 [29], Odc1 [20], Spsb1 [56], Crem [46], Sptlc2 [5], and Abca1 [77].

**Fig. 7:**
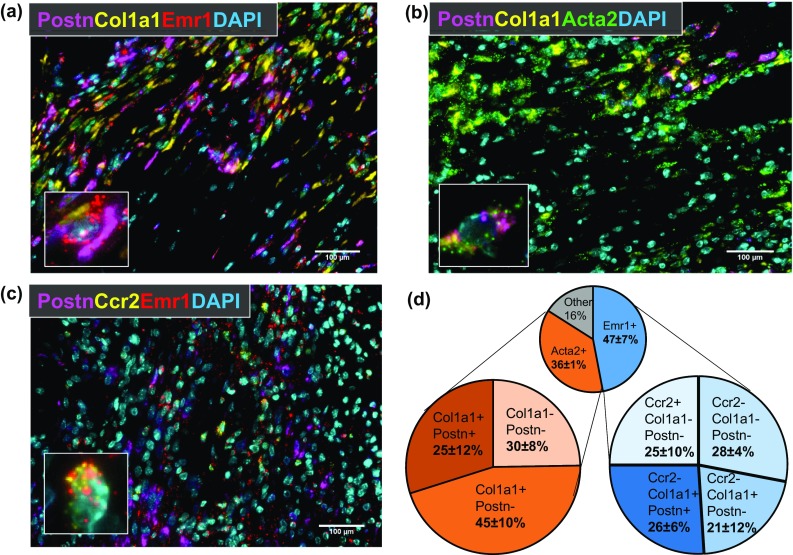
Macrophage expression of Col1a1 and Postn in LV infarct region. Numbers of cells expressing *Postn* and *Col1a1* mRNA in LV infarct tissue at day 7 post-MI were determined by in situ hybridization. Both *Emr1* (**a**; red, Cy5, macrophages) and *Acta2* (**b**; green, Cy5, fibroblasts) positive cells were assessed for expression of *Col1a1* (yellow, Cy3) and *Postn* (magenta, FITC). *Emr1* and *Ccr2* positive cells (**c**; yellow, Cy3) were assessed for expression of *Postn* (magenta, FITC). **d** Pie charts displaying proportion of LV infarct *Emr1 *+ (macrophages) and *Acta2 *+ (fibroblasts) cells expressing *Col1a1* and *Postn*. Nuclei are stained with DAPI (cyan)

Of note, Col1a1, Col3a1, and Postn, which contribute to post-MI scar formation and ECM stiffness, were all elevated in day 7 macrophages, while Col4a4 and Lama2, which contribute to basement membrane formation, and Mmp15, were decreased. Using triple in situ hybridization, we assessed the number of Emr1 + cells (i.e., macrophages), Acta2 + cells (i.e., fibroblasts), and Emr1 + Ccr2 + cells (infiltrating monocytes and macrophages) expressing Col1a1 and Postn mRNA in the infarct region of LV tissue from day 7 post-MI mice (Fig. 7a–c). Macrophages in the infarct region heterogeneously expressed both Col1a1 and Postn mRNA (Fig. 7d). Of the Emr1 + macrophages, about half expressed Col1a1 and about a quarter expressed Postn. ECM gene expression was found only in Ccr2 + cells, which have been shown to drive post-MI inflammation and impair ECM formation [45]. The majority of Acta2 + cells expressed Col1a1 (70%), while 25% also expressed Postn.

### Defining macrophage polarization phenotypes

In addition to the prototypical M1 and M2 markers, we investigated the most prominent genes uniquely upregulated at each post-MI day (Figs. 3c, 5d, 6c). Genes expressed only at day 0 (i.e., no or very low expression at all post-MI days) were ranked to assess day 0 (resident cardiac macrophage) markers. Day 0 genes were ranked by FPKM then ANOVA p value or by ANOVA p value then FPKM (Supplemental Table 3). The top ranked day 0 genes by FPKM included Atf3 [23], Cbr2, Folr2 [42, 58], Actr3 [90], and Cd81 [76]. The top ranked day 0 genes by p value included Cfh [2], Lilra5 [48], Cd209f [69], Cmah [60], and Tln2 [66].

As cell specificity is a good criterion for a marker, we compared the ranked genes to the literature to determine whether genes were distinctly expressed in macrophages relative to other cardiac MI-relevant cell types (myocytes, endothelial cells, fibroblasts, neutrophils, and lymphocytes). While several genes are ubiquitously expressed across cell types (Atf3, Cd81, Cmah, Tln2, Orm1, Cd9, Tnip1, Sarm1, Dek, Dnajc15, Tmsb4x, Rab18, Vdac1, and Odc1), there were genes with potential macrophage restricted expression (Cbr2, Folr2, Cfh, Lilra5, Cd209, Pcdha4, Slfn4, Tdgf1, Pcdha7, Slc3a1, Spsb1, and Abca1). Based on these criteria, Table 1 lists candidate markers that may uniquely identify cardiac macrophages from each time after MI, including directed analysis of known M1, M2, and macrophage markers and unbiased evaluation of the top ranked genes for each time examined.

**Table 1:** Candidate post-MI macrophage markers

	Day 0	Day 1	Day 3	Day 7
Informed	Ccl17, Retnla, Cd163	Il1b, Nos2, Arg1, Chi3l3, Cd14^a^, Lgals3^a^, Vegfa^a^	Ifng, Il12a, Lgals3^a^, Vegfa^a^	Cd14, Sparc, Lox, Lgals3^a^, Vegfa^a^
Unbiased	Cbr2, Folr2, Cfh, Lilra5, Cd209f	Pcdha4, Slfn4, Apobr, Gpr132	Klra22, Tdgf1, Il24	Spsb1, Abca1, Pcdha7, Klra23, Tac4, Slc3a1

Unbiased candidates are genes based on our ranking system; informed candidates are genes previously used as macrophage markers

^a^Marker for more than one time point

We assessed expression of genes involved in circadian rhythm regulation, as time of surgery and cell isolation all were performed between 8 a.m. and 12 noon (Supplemental Fig. 8). No differences in Clock, Per2, or Nr1d1 were observed, while Nr1d2, Nfil3, Arnt1, and Cry1 were differentially expressed after MI. These results indicate that MI may compromise circadian rhythm regulation in macrophages, which has been implicated in release of cytokines and other macrophage functions [84].

## Discussion

The goal of this study was to map the continuum of changes that occur in cardiac macrophages over the first week of MI. The key findings were: (1) cardiac macrophages undergo continual and distinct transcriptomic changes at post-MI days 1, 3, and 7; and (2) day 1 macrophages have a pro-inflammatory profile, day 3 macrophages have a phagocytic, proliferative, and metabolic reprogramming profile, and day 7 macrophages have a reparative signature that includes expression of extracellular matrix remodeling genes that contribute to scar formation (e.g., collagen 1a1 and periostin). While macrophages in the post-MI heart have been characterized in terms of cell surface markers, phenotypes, and origins, the full transcriptome has not been mapped in detail [9, 12–14, 18, 22].

Macrophages underwent distinct gene expression changes reflecting shifts in phenotype over the first week of post-MI LV remodeling. The fact that day 7 normalized values showed distinction in expression from day 3 normalized values (e.g., Col1a1, Col3a1, and Postn genes) indicates there is still a continuum of changes occurring, at a slower kinetic rate than day 0 through days 1 and 3. Principal component analysis indicates that day 1 macrophages are markedly different in phenotype than all other time points, whereas day 3 and 7 macrophages are closer to a day 0 phenotype, indicating the transition to a new homeostatic-like phenotype.

Consistent with the literature, our results indicate that the early day 1 post-MI macrophage shows a strong pro-inflammatory, matrix-degrading phenotype [22]. In addition to pro-inflammation, enrichment analysis indicated that glycolysis and cellular response to hypoxia were upregulated in the day 1 macrophage, suggesting an adaptation to the hypoxic environment of the early infarct. Hypoxia induces activation of the hypoxia-inducible factor (HIF)-1α pathway, which turns on pro-inflammatory gene expression, as well as metabolic reprogramming towards glycolysis [1, 9]. Hif1a was high in day 1 and 3 post-MI macrophages and returned towards day 0 values by day 7. While day 1 macrophages upregulate pro-inflammatory genes early in response to MI, several genes that inhibit these pathways are also highly induced (e.g., Slfn4, Cd9, Tnip1, and Gpr132), most likely to exert negative feedback and limit excessive inflammation.

Macrophage metabolism is a reflection of and also a contributor to polarization status, as pro-inflammatory M1 macrophages rely on glycolysis, while reparative M2 macrophages use oxidative phosphorylation [33, 57]. Indeed, our results show that by day 3 post-MI, genes related to mitochondrial ATP generation and oxidative phosphorylation were upregulated, indicating metabolic reprogramming over the post-MI remodeling continuum. While studies have demonstrated that metabolism influences macrophage polarization, the role of metabolism in post-MI macrophage polarization has not been extensively evaluated [16, 33]. Our results indicate the metabolic shift of the day 3 macrophage may be an indicator of wound repair status.

At day 3 post-MI, macrophages downregulate many of the inflammatory genes elevated at day 1, including Il1b, while upregulating others, including Il12a, Pf4, and Il24. In addition, day 3 macrophages showed increased phagocytic capacity and a return in proliferation. Phagocytosis of necrotic and apoptotic cells is a critical role of post-MI macrophages and is required for the transition from a pro- to anti-inflammatory environment and polarization towards a reparative phenotype [18, 32, 65]. Resident cardiac macrophages have a basal phagocytic rate, while day 1 MI macrophages have increased phagocytic capacity [22, 54]. Our results indicate that phagocytic capacity is elevated at day 3, which coincides with downregulation of many of the inflammatory genes that were elevated at day 1. Following MI, macrophage phagocytosis occurs in two sequential steps: initial phagocytosis of necrotic myocytes, followed by efferocytosis of apoptotic neutrophils [68]. The increased phagocytic capacity that we observed at day 3 post-MI may represent efferocytosis of apoptotic neutrophils, whose numbers in the infarct concomitantly begin to decline after day 3. As resident cardiac macrophages are proliferative, the similarity in proliferation rates between day 0 and day 3 macrophages indicates that by day 3 proliferation of macrophages is re-initiated, consistent with a previous report [22].

At day 7 post-MI, macrophages exhibited a reparative phenotype, indicated by upregulation of ECM organization genes. Interestingly, genes typically considered fibroblast-specific were upregulated in day 7 macrophages, including Col1a1 and Postn. Recent studies on aging hearts have characterized a myeloid-derived cardiac fibroblast population, which is derived from M2a macrophages [81]. Our study suggests that post-MI macrophages may assume a fibroblast-like phenotype, which remains to be fully investigated. While macrophages indirectly contribute to ECM formation by releasing paracrine factors that stimulate cardiac fibroblasts, macrophages can directly secrete ECM proteins [6]. Indeed, fibronectin expression was highly upregulated in day 1 macrophages and returned towards baseline by day 7. Collagens type VI and VIII were also elevated at day 7 post-MI and have been shown to increase in response to anti-inflammatory stimuli (e.g., TGF-β1 and IL-4) and decrease in response to pro-inflammatory stimuli (e.g., LPS and IFN-γ) [71, 89]. Our results reveal a previously undefined role for macrophages in directly contributing ECM proteins to the infarct scar.

Macrophage polarization towards M1 and M2 subtypes has been well-defined in vitro for some, but not all stimuli. Post-MI in vivo macrophage polarization is less clear, as there are a number of factors over a broad spectrum within the infarct environment that co-stimulate to generate an overall macrophage phenotype. After MI, the inflammatory M1 phenotype predominates early (day 1), whereas the M2 reparative phenotype later becomes the major subtype (day 7). While our enrichment analyses indicated a pro-inflammatory day 1 post-MI phenotype and an anti-inflammatory/reparative phenotype at day 7 post-MI, gene expression for typical M1 and M2 markers did not reflect the in vitro scenario. For example, while Nos2 (an M1 marker) was increased 5-fold at day 1, Arg1 (an M2 marker) was increased over 900-fold. Nos2 returned to baseline at days 3 and 7, while Arg1 remained elevated. The concept of arginase-1 as an M2 marker has been challenged, as a variety of M1 stimuli induce arginase-1 expression [27]. It is possible that ratios or combinations of profiles dictate net phenotype, with fine tuning occurring by fluxes in both pro- and anti-inflammatory constituents.

Our results indicate that IL-1β and IFN-γ are major macrophage-derived cytokines that promote the pro-inflammatory environment early post-MI, as Il6 was unchanged and Tnf was decreased. Il12a, which has previously been reported to be undetectable in the infarcted LV, was decreased in macrophages at day 1 post-MI, increased at day 3, and returned to day 0 values by day 7 [17]. Expressions of the anti-inflammatory cytokines Tgfb1 and Il10 were unchanged in macrophages following MI. One explanation is that other cell types are the major source of these cytokines; a second is that regulation occurs at the post-translational stage. Tgfb1 is regulated at the post-translational stage, as pre-formed TGFβ1 protein is produced and sequestered in the ECM by binding to latent TGFβ binding protein. Bioavailability is regulated by proteases (including MMPs) that cleave the binding protein. Ym1 (Chi3l3) and Fizz1 (Retnla) have been implicated as M2 macrophage markers [27, 67]. Ym1/Chi3l3, which was highly upregulated at days 1 and 3 post-MI and downregulated at day 7, is a lectin that binds heparan-type glycosaminoglycans to influence inflammation [69]. Fizz1/Retnla was highly decreased in macrophages at all days post-MI, and its role in inflammation is less clear, as it possesses both pro- and anti-inflammatory roles depending on context [69]. Our results support the growing consensus to abandon the M1 and M2 nomenclature, as pro-inflammatory day 1 macrophages do not fully display typical M1 features nor do pro-reparative day 7 macrophages display typical M2 features [53].

**Fig. 8:**
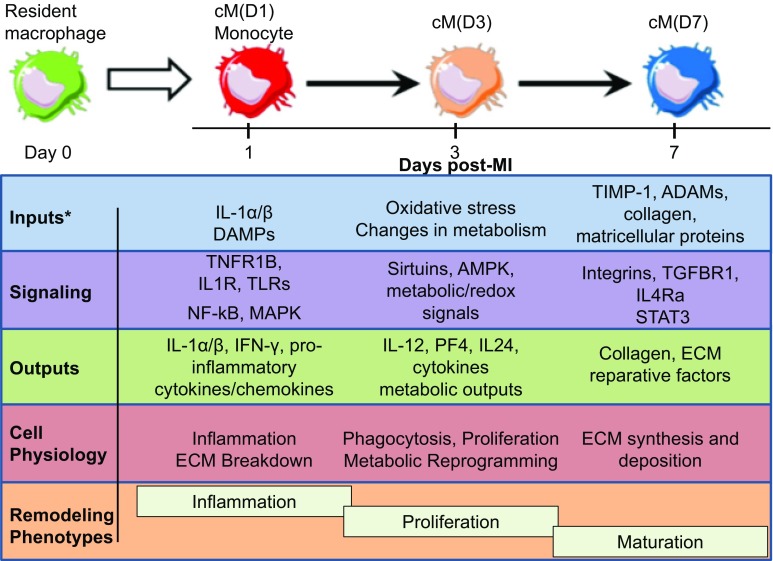
Map of macrophage polarization over the MI time continuum. Post-MI macrophage profiles in terms of inputs, signaling, outputs, and cell physiology over the three major post-MI remodeling phases (inflammation, proliferation, and scar maturation). *DAMPs* damage-associated molecular patterns, *ECM* extracellular matrix

The post-MI macrophage polarization time continuum map is shown in Fig. 8. Day 1 macrophages exhibit a NF-κB and MAPK signaling repertoire, which are activated in response to toll-like receptor signaling pathways and induce downstream pro-inflammatory molecules [27]. At day 3, the sirtuin and AMPK axis, which promote the shift from glycolysis to mitochondrial oxidative phosphorylation and polarization towards a reparative phenotype, may be important regulators of macrophage signaling [51, 86]. In day 7 macrophages, STAT3/4, TGFBR1, and IL4Ra were important pathways regulating the reparative phenotype. Both IL-6 and IL-10 can promote M2 polarization through STAT3 activation [85]. Signaling through TGFBR1 influences macrophage polarization by inhibiting TNF-α and iNOS expression and strongly induces collagen I and III expression in fibroblasts [8, 35, 72]. IL-4Ra, which was increased at day 7 post-MI, drives M2 polarization and is critical for macrophage regulation of collagen remodeling in skin wound healing [31]. Itga11, which was elevated at day 7 post-MI, encodes for a collagen-binding integrin that regulates intracellular collagen production [64].

Resident (day 0) cardiac macrophages are derived from a different source than infiltrating monocyte-derived macrophages. While the different origins of these two populations of macrophages could account for transcriptomic and phenotypic differences, the purpose of our study was to determine differences in macrophages within the cardiac environment that reflect cell physiological roles in maintenance and wound healing, rather than differences in circulating monocytes before MI compared to infiltrating tissue monocytes after MI. Further, differentially expressed genes were largely distinct across days 1, 3, and 7, indicating that the patterns of gene expression were not primarily reflecting cell source. Genes that were similarly differentially expressed across all three of these days may be related to either cell source or sustained expression. At last, the procedure of isolating cardiac macrophages is different from isolating circulating monocytes, which could complicate interpretation of gene expression data.

Transcriptomic analysis was performed on all cardiac macrophages, rather than focusing on distinct macrophage subpopulations. As this study is the first to report distinct changes in the macrophage transcriptome along the MI time course, our study provides the necessary framework for future studies to analyze distinct macrophage subpopulations within each time point. There was no indication that pooling increased variation, as the coefficient of variation (CV) for individual cell counts was the smallest at day 7 post-MI, whereas the CV for genes in Cluster 3 (Col1a1 and Postn) was highest at the time, indicating biological variability in response. Previous studies in our lab have observed biological variation in expression of these genes (Col1a1, CV = 140% and Postn, CV = 148%) in day 7 post-MI macrophages isolated from individual mice [28]. In the present study, CV was 41% for Col1a1 and 46% for Postn, indicating there was no evidence of increased variation due to pooling.

Our study focused only on surviving mice, which raises the question of changes in the macrophage transcriptome in surviving mice versus mice that undergo cardiac rupture. Although fresh cardiac tissue is difficult to obtain from mice with cardiac rupture due to its spontaneous nature, serum factors (i.e., factor XIII) or circulating monocyte markers could be used to predict survival versus rupture [52].

In conclusion, macrophages show distinct transcriptomic profiles at different time points over the first week of post-MI wound healing. Metabolic shifts in post-MI macrophages may regulate polarization and cell physiology status and remain to be fully investigated. Of note, macrophages may play a more direct role in post-MI ECM synthesis and remodeling than previously thought. Our findings provide novel insights into the potential mechanisms and pathways that regulate macrophage physiology during the inflammatory, granulation, and maturation phases of post-MI cardiac remodeling. Overall, our work suggests that attention to temporal profiles should be given when considering therapeutic strategies that alter macrophages.

## Electronic supplementary material

Below is the link to the electronic supplementary material.

Supplementary material 1 (DOCX 14 kb)

Supplementary material 2 (PPTX 154 kb)

Supplementary material 3 (PPTX 244 kb)

Supplementary material 4 (PPTX 330 kb)

Supplementary material 5 (PPTX 397 kb)

Supplementary material 6 (PPTX 758 kb)

Supplementary material 7 (PPTX 161 kb)

Supplementary material 8 (PPTX 265 kb)

Supplementary material 9 (PPTX 260 kb)

Supplementary material 10 (XLSX 2700 kb)

Supplementary material 11 (DOCX 10 kb)

Supplementary material 12 (DOCX 12 kb)
