# Supplementary material for: Mapping macrophage polarization over the myocardial infarction time continuum
Source: Basic Res Cardiol. 2018 Jun 4;113(4):26. doi: 10.1007/s00395-018-0686-x (PMC5986831; doi:10.1007/s00395-018-0686-x)
Supplement: Supplementary file 1 — Supplementary material 1 (DOCX 14 kb) [file 395_2018_686_MOESM1_ESM.docx]

**Supplemental Material**

**Supplemental Figure 1. RNA-Seq Comparison of Day 3 post-MI Macrophages Cultured for 2 h versus 20 h.** (a) Heat map analysis for differentially expressed genes (fold change>2 and p<0.05). Culturing for 20 h increased expression of 1522 genes and decreased 1947 genes compared to 2 h incubation. (b) Top 5 biological processes and combined scores for upregulated and downregulated genes in EnrichR.

**Supplemental Figure 2. Evidence of MI.** MI resulted in the expected decrease in infarct wall thickness, increase in LV dilation, and decrease in cardiac physiology at MI days 1, 3, and 7. (a) 7-day survival. (b) Infarct area calculated by LV infarct mass to total mass. MI areas at day 1, 3, and 7 were similar. (c) MI resulted in decreased infarct wall thickness at all days, (d) increased end-diastolic and (e) end-systolic dimensions at days 1 and 3 and further increased at day 7, and (f) decreased fractional shortening at all times. *p<0.05 vs. day 0, #p<0.05 vs. day 1, $p<0.05 vs. day 3.

**Supplemental Figure 3. Cell Purity Assessment.** Top: FPKM values for cardiac cell specific markers (macrophage, endothelial, fibroblast, lymphocyte, myocyte, and neutrophil) across the post-MI time course. Bottom: Magnification of the macrophage markers. *p<0.05 vs. day 0, #p<0.05 vs. day 1, $p<0.05 vs. day 3.

**Supplemental Figure 4. M1 and M2 Markers.** Expression values for common M1 and M2 macrophage polarization markers. *p<0.05 vs. day 0, #p<0.05 vs. day 1, $p<0.05 vs. day 3.

**Supplemental Figure 5. RT-PCR Validation of RNA-Seq.** (Left Column) RT-PCR, 2^-ΔCt^ values. (Middle Column) FPKM values. (Right Column) Pearson’s correlation between 2^-ΔCt^ values and FPKM values. *p<0.05 vs. day 0, #p<0.05 vs. day 1, $p<0.05 vs. day 3.

**Supplemental Figure 6. IPA Enrichment Analyses of Individual Post-MI Days.** Upregulated and downregulated processes at post-MI day 1, 3, and 7.

**Supplemental Figure 7. Genes Involved in Phagocytosis and Proliferation.** *p<0.05 vs. day 0, #p<0.05 vs. day 1, $p<0.05 vs. day 3.

**Supplemental Figure 8. Circadian Rhythm Genes.** FPKM values of genes associated with circadian rhythm. *p<0.05 vs. day 0, #p<0.05 vs. day 1, $p<0.05 vs. day 3.

**Supplemental Table 1.** FPKM values for all 16,695 genes included in the dataset.

**Supplemental Table 2.** Extracellular matrix (ECM) Genes Upregulated and Downregulated at day 7.

**Supplemental Table 3.** Day 0 macrophage markers.
