# Supplementary material for: Mapping macrophage polarization over the myocardial infarction time continuum
Source: Basic Res Cardiol. 2018 Jun 4;113(4):26. doi: 10.1007/s00395-018-0686-x (PMC5986831; doi:10.1007/s00395-018-0686-x)
Supplement: Supplementary file 2 — Supplementary material 2 (PPTX 154 kb) [file 395_2018_686_MOESM2_ESM.pptx]

## Slide 1
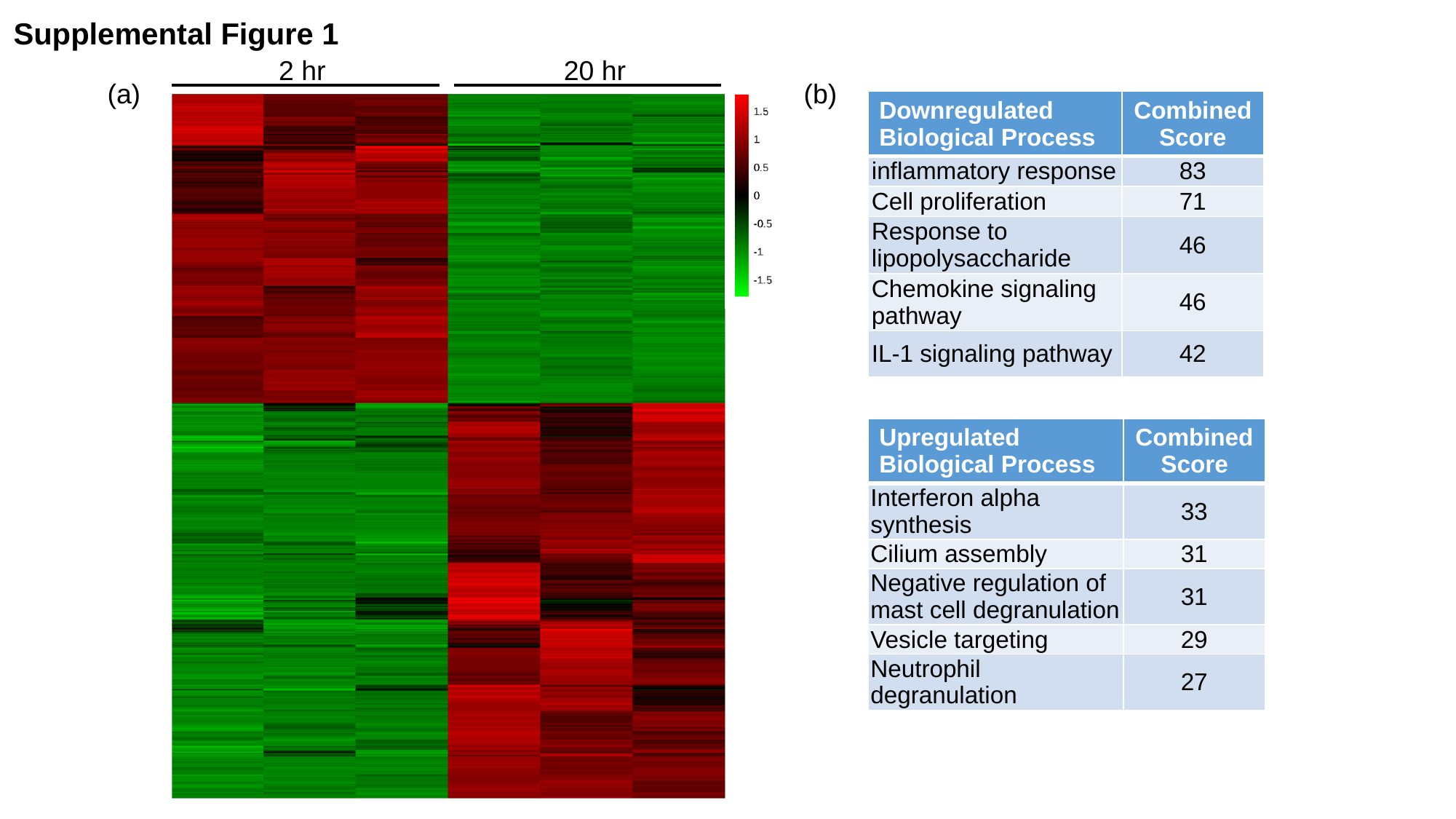

Supplemental Figure 1
2 hr
20 hr
(a)
(b)
| Downregulated Biological Process | Combined Score |
| --- | --- |
| inflammatory response | 83 |
| Cell proliferation | 71 |
| Response to lipopolysaccharide | 46 |
| Chemokine signaling pathway | 46 |
| IL-1 signaling pathway | 42 |
| Upregulated Biological Process | Combined Score |
| --- | --- |
| Interferon alpha synthesis | 33 |
| Cilium assembly | 31 |
| Negative regulation of mast cell degranulation | 31 |
| Vesicle targeting | 29 |
| Neutrophil degranulation | 27 |
