# Supplementary material for: Mapping macrophage polarization over the myocardial infarction time continuum
Source: Basic Res Cardiol. 2018 Jun 4;113(4):26. doi: 10.1007/s00395-018-0686-x (PMC5986831; doi:10.1007/s00395-018-0686-x)
Supplement: Supplementary file 3 — Supplementary material 3 (PPTX 244 kb) [file 395_2018_686_MOESM3_ESM.pptx]

## Slide 1
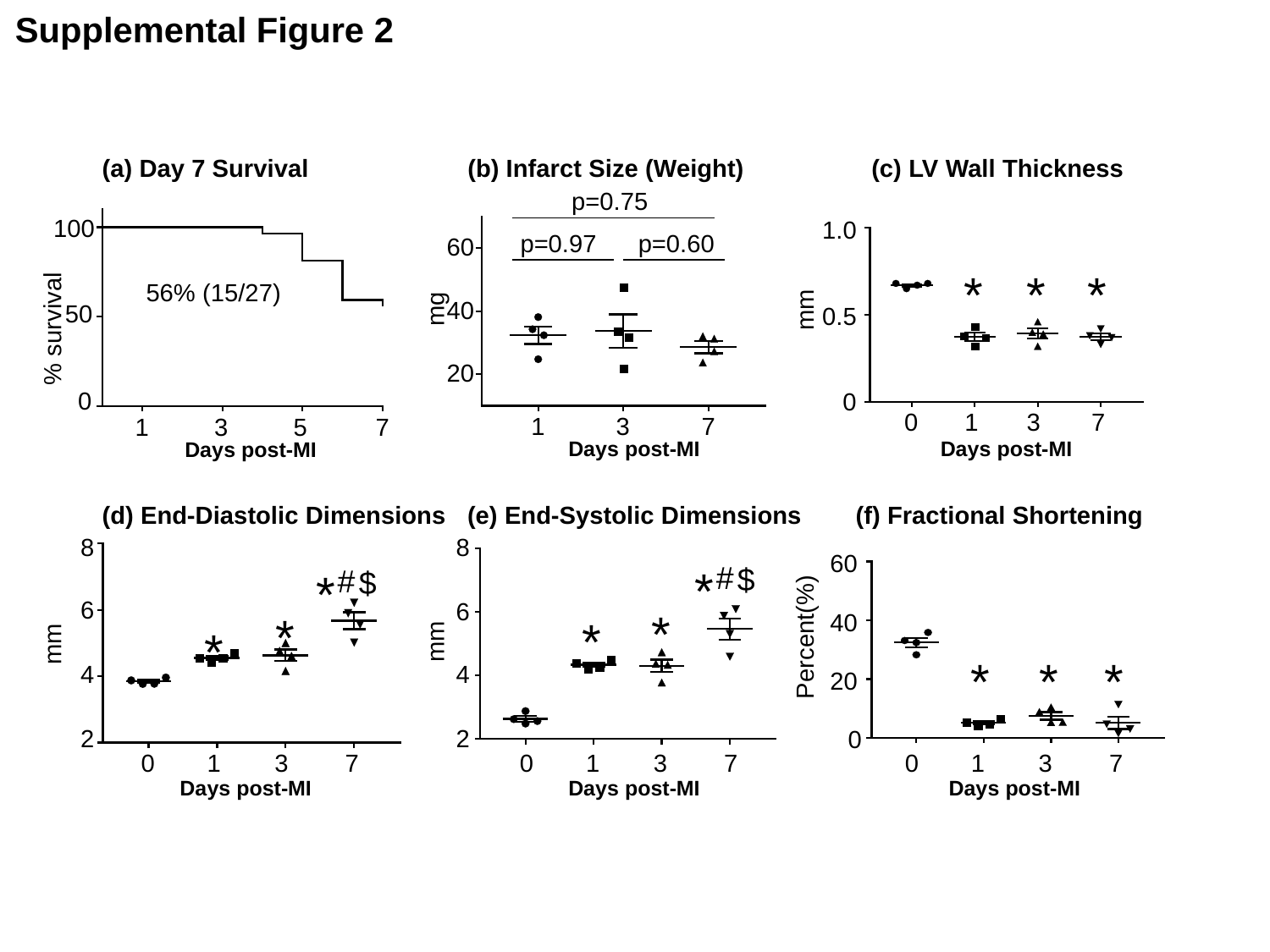

Supplemental Figure 2
(a) Day 7 Survival
(b) Infarct Size (Weight)
(c) LV Wall Thickness
p=0.75
100
1.0
p=0.97
p=0.60
60
*
*
*
56% (15/27)
% survival
40
mg
50
mm
0.5
20
0
0
0
1
3
7
1
3
7
1
3
5
7
Days post-MI
Days post-MI
Days post-MI
(d) End-Diastolic Dimensions
(e) End-Systolic Dimensions
(f) Fractional Shortening
8
8
60
#
*
$
#
*
$
Percent(%)
6
6
*
*
40
*
mm
mm
*
*
*
*
4
4
20
2
2
0
0
1
3
7
0
1
3
7
0
1
3
7
Days post-MI
Days post-MI
Days post-MI
