# Supplementary material for: Mapping macrophage polarization over the myocardial infarction time continuum
Source: Basic Res Cardiol. 2018 Jun 4;113(4):26. doi: 10.1007/s00395-018-0686-x (PMC5986831; doi:10.1007/s00395-018-0686-x)
Supplement: Supplementary file 4 — Supplementary material 4 (PPTX 330 kb) [file 395_2018_686_MOESM4_ESM.pptx]

## Slide 1
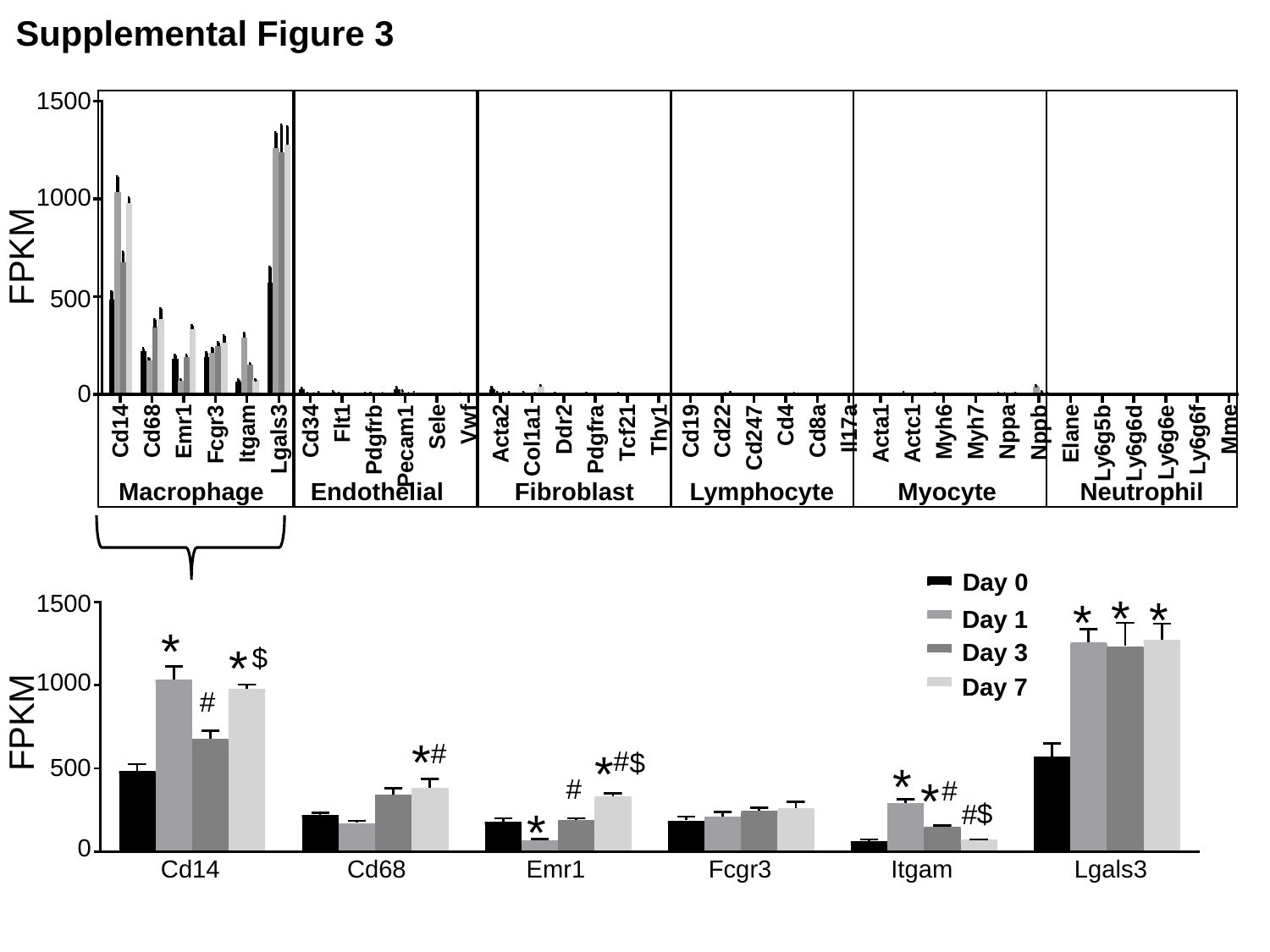

Supplemental Figure 3
1500
1000
FPKM
500
0
Macrophage
Endothelial
Fibroblast
Lymphocyte
Myocyte
Neutrophil
Day 0
*
1500
*
*
Day 1
*
*
Day 3
$
1000
Day 7
#
FPKM
*
#
*
#
$
500
*
*
#
#
$
#
*
0
Cd14
Cd68
Emr1
Fcgr3
Itgam
Lgals3
