# Supplementary material for: Mapping macrophage polarization over the myocardial infarction time continuum
Source: Basic Res Cardiol. 2018 Jun 4;113(4):26. doi: 10.1007/s00395-018-0686-x (PMC5986831; doi:10.1007/s00395-018-0686-x)
Supplement: Supplementary file 5 — Supplementary material 5 (PPTX 397 kb) [file 395_2018_686_MOESM5_ESM.pptx]

## Slide 1
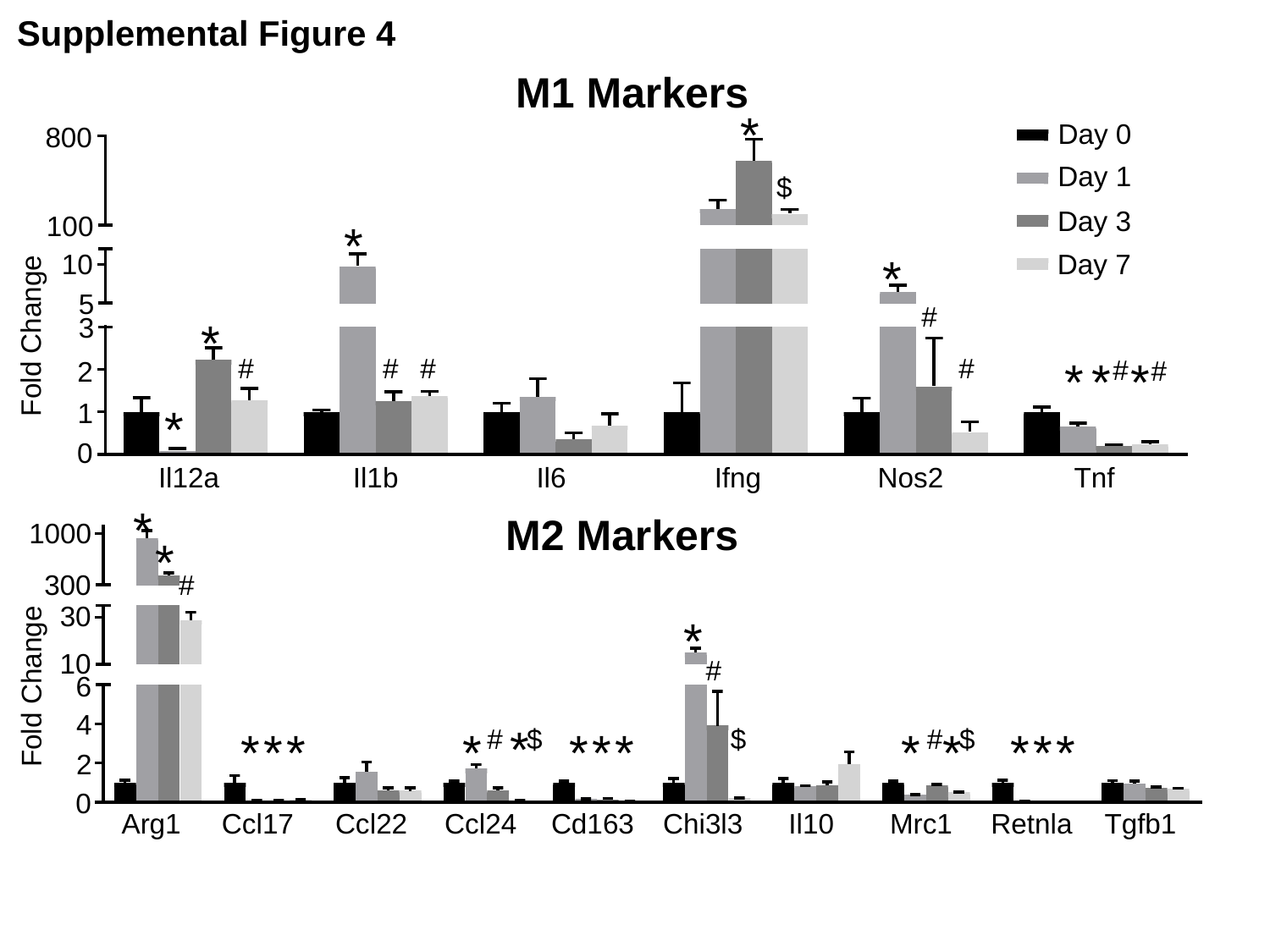

Supplemental Figure 4
M1 Markers
*
Day 0
800
Day 1
$
Day 3
100
*
10
*
Day 7
5
#
Fold Change
3
*
#
#
#
#
*
*
*
#
#
2
1
*
0
Il12a
Il1b
Il6
Ifng
Nos2
Tnf
*
M2 Markers
1000
*
300
#
30
*
10
#
Fold Change
6
4
*
*
*
*
*
#
$
*
*
*
$
*
#
*
$
*
*
*
2
0
Chi3l3
Il10
Mrc1
Retnla
Tgfb1
Cd163
Arg1
Ccl17
Ccl22
Ccl24
