# Supplementary material for: Mapping macrophage polarization over the myocardial infarction time continuum
Source: Basic Res Cardiol. 2018 Jun 4;113(4):26. doi: 10.1007/s00395-018-0686-x (PMC5986831; doi:10.1007/s00395-018-0686-x)
Supplement: Supplementary file 6 — Supplementary material 6 (PPTX 758 kb) [file 395_2018_686_MOESM6_ESM.pptx]

## Slide 1
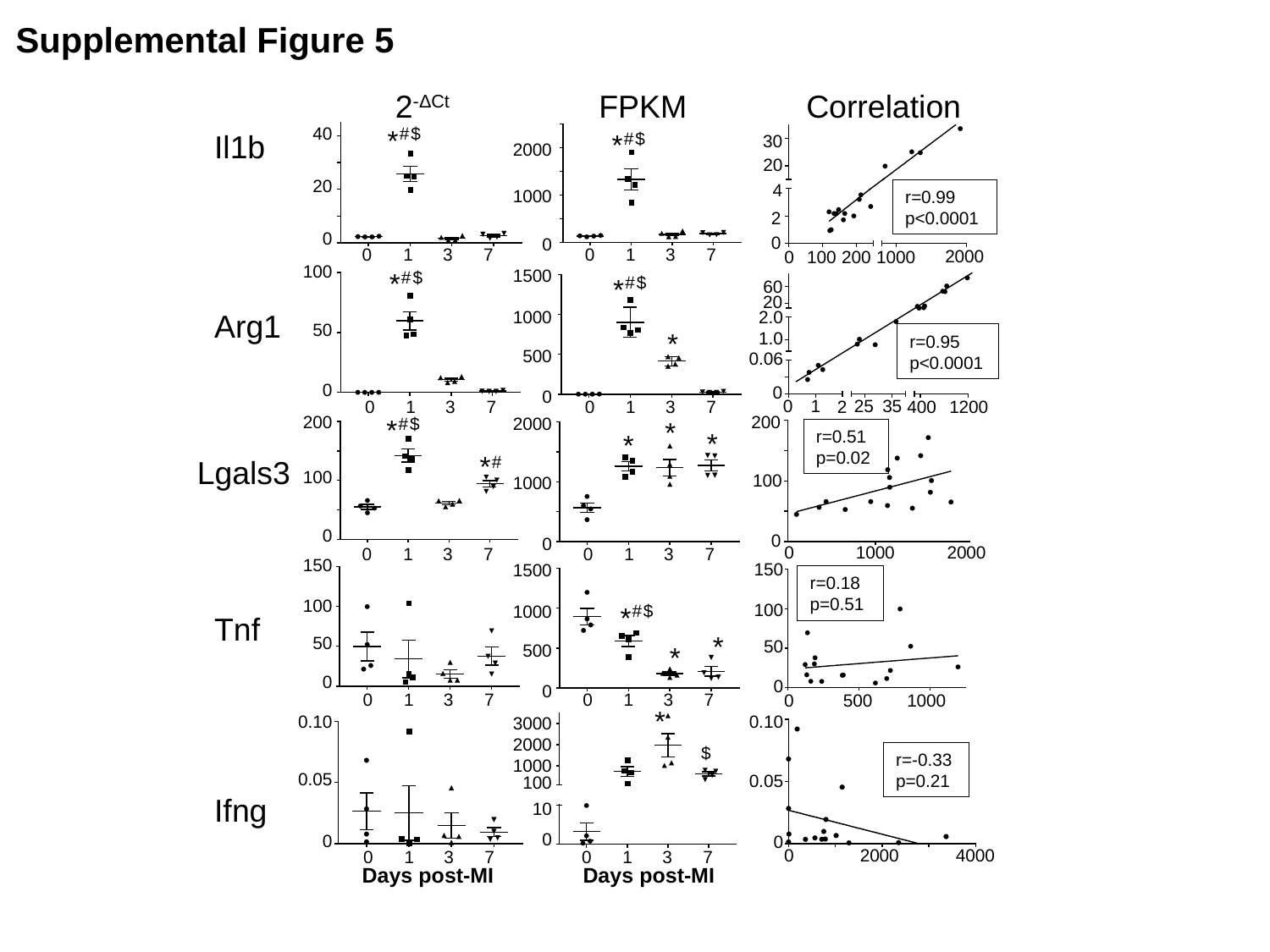

Supplemental Figure 5
2-ΔCt
FPKM
Correlation
*
#
$
Il1b
*
#
$
r=0.99
p<0.0001
*
#
$
*
#
$
Arg1
*
r=0.95
p<0.0001
*
#
$
*
r=0.51
p=0.02
*
*
*
#
Lgals3
r=0.18
p=0.51
*
#
$
Tnf
*
*
*
$
r=-0.33
p=0.21
Ifng
Days post-MI
Days post-MI
40
30
2000
20
20
4
1000
2
0
0
0
0
1
3
7
0
1
3
7
2000
0
100
200
1000
100
1500
60
20
1000
2.0
50
1.0
500
0.06
0
0
0
0
1
25
35
0
1
3
7
0
1
3
7
2
400
1200
200
200
2000
100
100
1000
0
0
0
0
1000
2000
0
1
3
7
0
1
3
7
150
150
1500
100
100
1000
50
50
500
0
0
0
0
1
3
7
0
1
3
7
0
500
1000
0.10
0.10
3000
2000
1000
0.05
0.05
100
10
0
0
0
0
2000
4000
0
1
3
7
0
1
3
7
