# Supplementary material for: Mapping macrophage polarization over the myocardial infarction time continuum
Source: Basic Res Cardiol. 2018 Jun 4;113(4):26. doi: 10.1007/s00395-018-0686-x (PMC5986831; doi:10.1007/s00395-018-0686-x)
Supplement: Supplementary file 7 — Supplementary material 7 (PPTX 161 kb) [file 395_2018_686_MOESM7_ESM.pptx]

## Slide 1
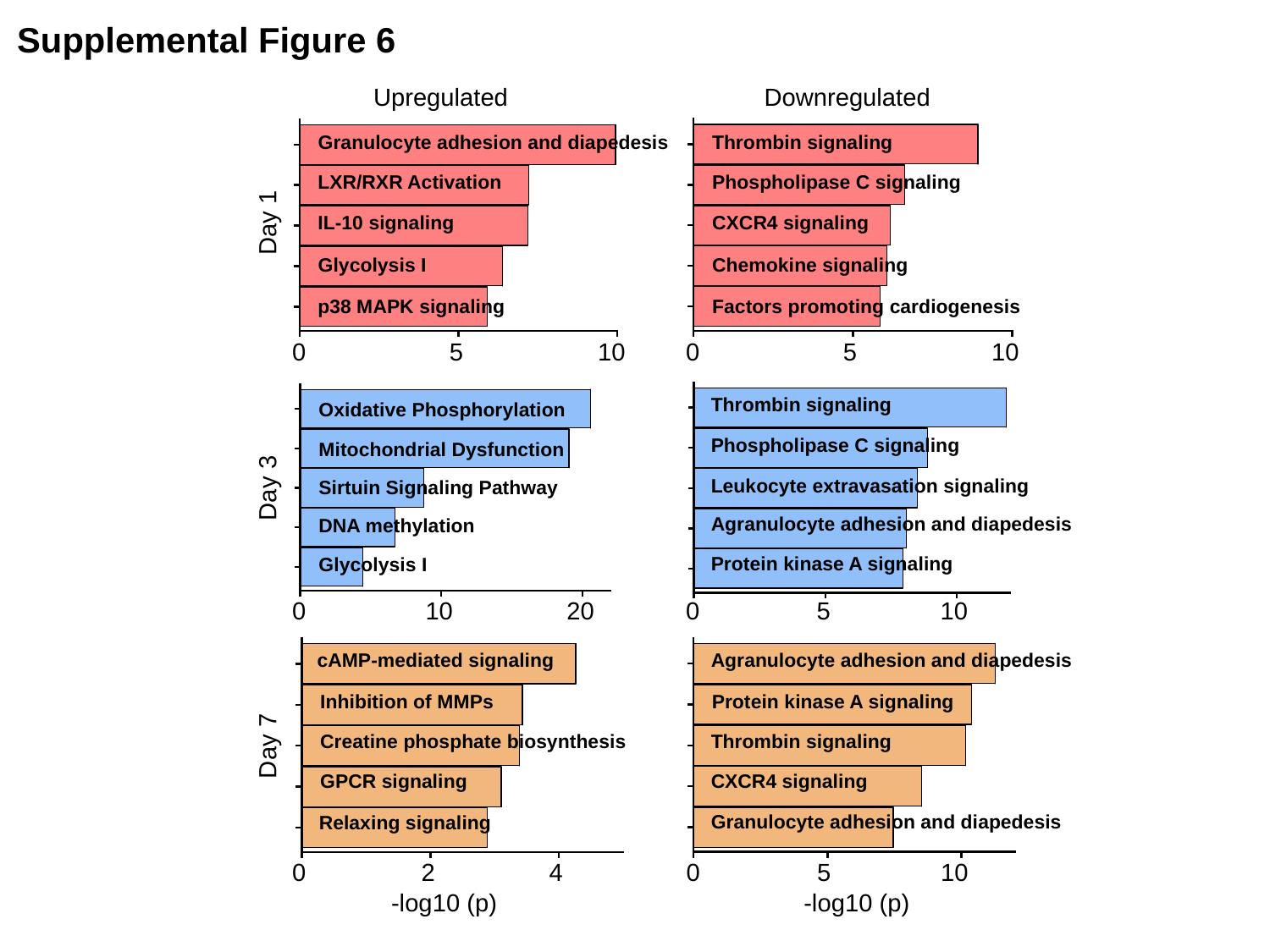

Supplemental Figure 6
Upregulated
Downregulated
Granulocyte adhesion and diapedesis
Thrombin signaling
LXR/RXR Activation
Phospholipase C signaling
Day 1
IL-10 signaling
CXCR4 signaling
Glycolysis I
Chemokine signaling
p38 MAPK signaling
Factors promoting cardiogenesis
0
5
10
0
5
10
Thrombin signaling
Oxidative Phosphorylation
Phospholipase C signaling
Mitochondrial Dysfunction
Day 3
Leukocyte extravasation signaling
Sirtuin Signaling Pathway
Agranulocyte adhesion and diapedesis
DNA methylation
Protein kinase A signaling
Glycolysis I
0
10
20
0
5
10
cAMP-mediated signaling
Agranulocyte adhesion and diapedesis
Inhibition of MMPs
Protein kinase A signaling
Day 7
Creatine phosphate biosynthesis
Thrombin signaling
GPCR signaling
CXCR4 signaling
Granulocyte adhesion and diapedesis
Relaxing signaling
0
2
4
0
5
10
-log10 (p)
-log10 (p)
