# Supplementary material for: Mapping macrophage polarization over the myocardial infarction time continuum
Source: Basic Res Cardiol. 2018 Jun 4;113(4):26. doi: 10.1007/s00395-018-0686-x (PMC5986831; doi:10.1007/s00395-018-0686-x)
Supplement: Supplementary file 8 — Supplementary material 8 (PPTX 265 kb) [file 395_2018_686_MOESM8_ESM.pptx]

## Slide 1
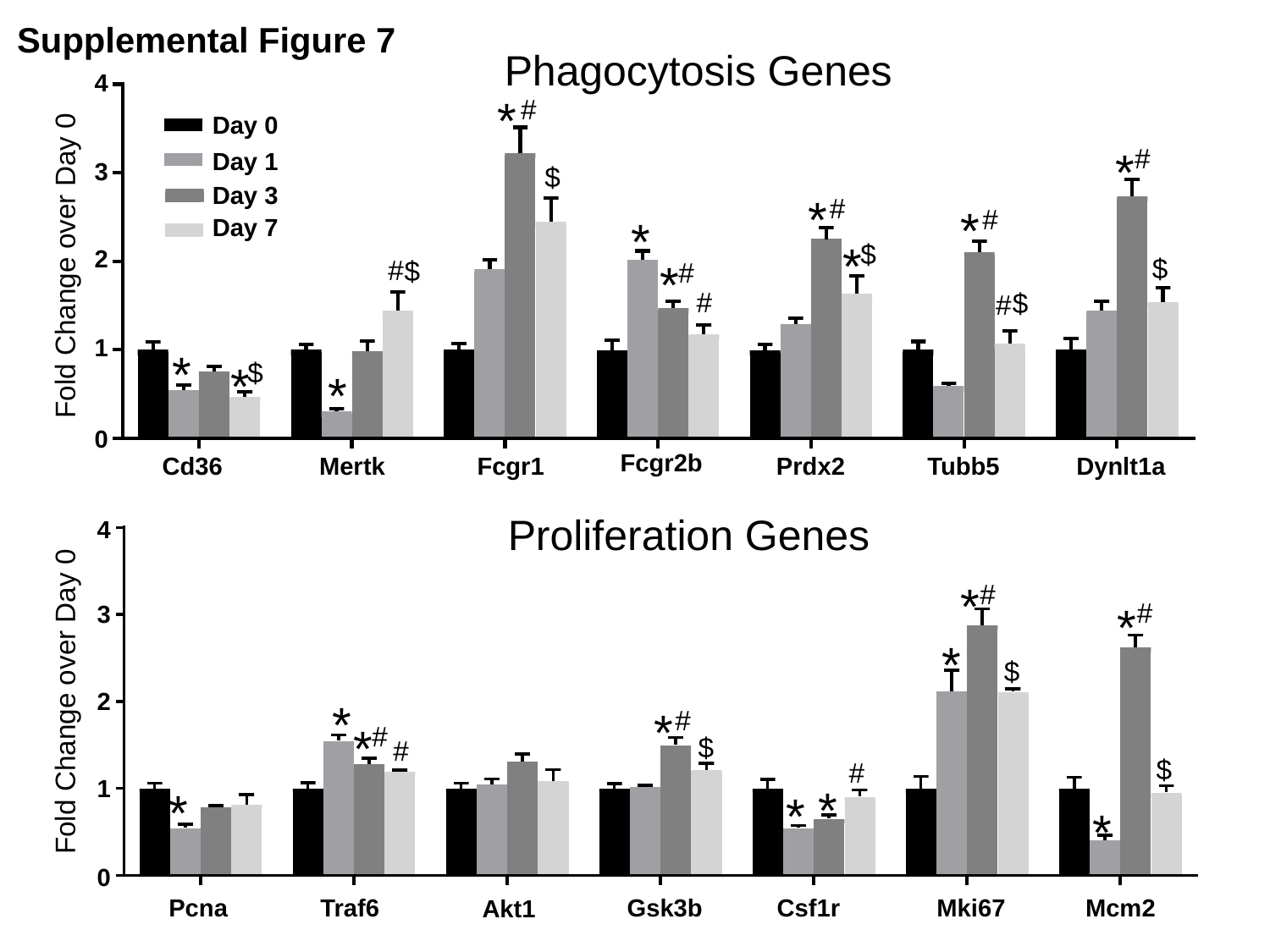

Phagocytosis Genes
*
*
$
Fcgr2b
Cd36
Mertk
Fcgr1
Prdx2
Dynlt1a
Tubb5
*
#
*
#
$
*
#
*
#
*
*
$
$
#
*
$
#
#
$
#
*
Supplemental Figure 7
4
Day 0
Day 1
3
Fold Change over Day 0
Day 3
Day 7
2
1
0
Proliferation Genes
*
#
*
#
*
$
*
*
#
*
#
$
#
$
#
*
*
*
*
Mki67
Mcm2
Csf1r
Gsk3b
Pcna
Traf6
Akt1
4
3
Fold Change over Day 0
2
1
0
