# Supplementary material for: Mapping macrophage polarization over the myocardial infarction time continuum
Source: Basic Res Cardiol. 2018 Jun 4;113(4):26. doi: 10.1007/s00395-018-0686-x (PMC5986831; doi:10.1007/s00395-018-0686-x)
Supplement: Supplementary file 9 — Supplementary material 9 (PPTX 260 kb) [file 395_2018_686_MOESM9_ESM.pptx]

## Slide 1
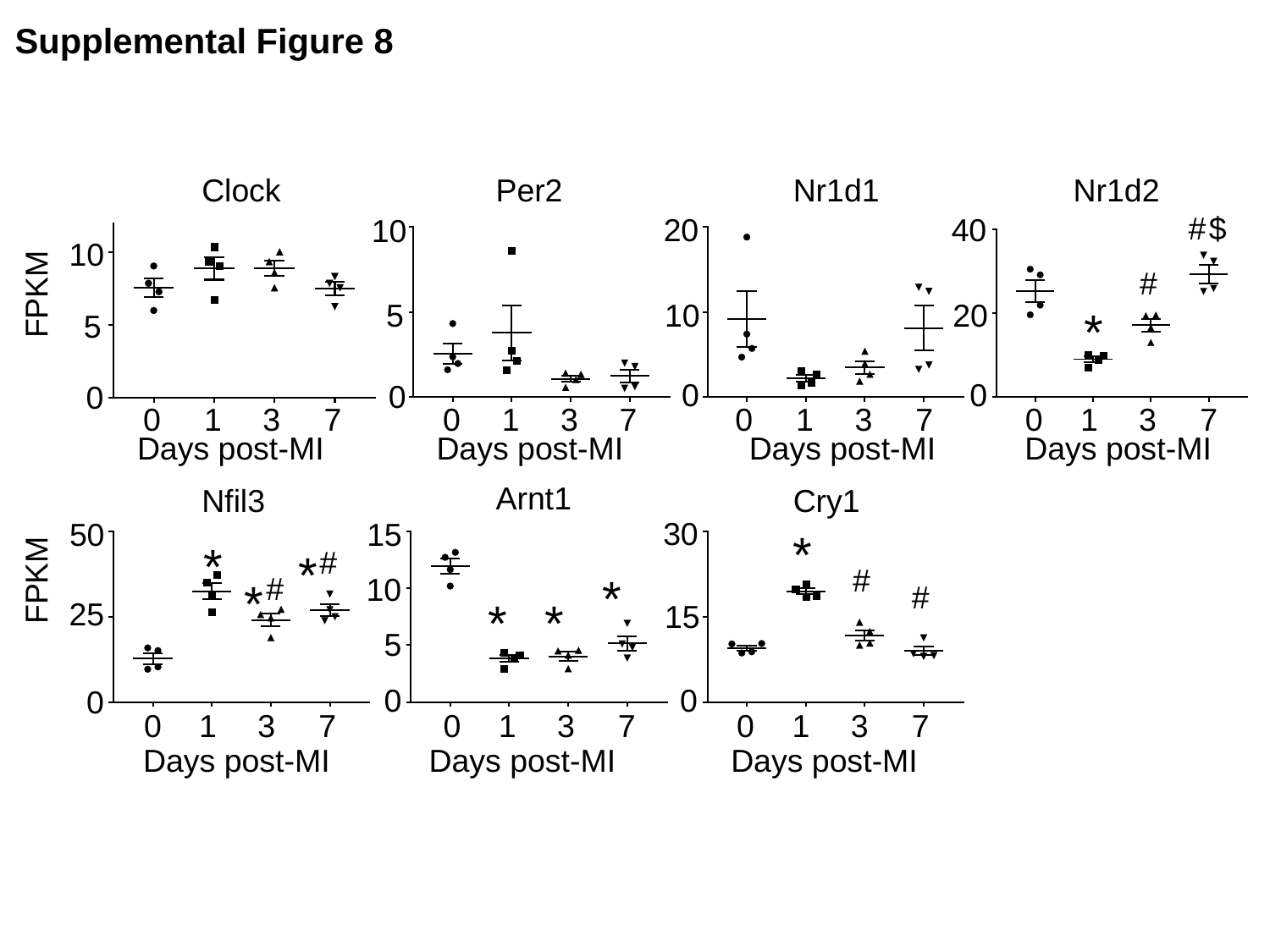

Supplemental Figure 8
Clock
Per2
Nr1d1
Nr1d2
#
$
20
40
10
10
#
FPKM
5
10
20
*
5
0
0
0
0
0
1
3
7
0
1
3
7
0
1
3
7
0
1
3
7
Days post-MI
Days post-MI
Days post-MI
Days post-MI
Arnt1
Nfil3
Cry1
30
50
15
*
*
#
*
FPKM
#
*
#
10
*
#
*
*
25
15
5
0
0
0
0
1
3
7
0
1
3
7
0
1
3
7
Days post-MI
Days post-MI
Days post-MI
