# Supplementary material for: Mapping macrophage polarization over the myocardial infarction time continuum
Source: Basic Res Cardiol. 2018 Jun 4;113(4):26. doi: 10.1007/s00395-018-0686-x (PMC5986831; doi:10.1007/s00395-018-0686-x)
Supplement: Supplementary file 11 — Supplementary material 11 (DOCX 10 kb) [file 395_2018_686_MOESM11_ESM.docx]

| **Supplemental Table 2.** ECM Genes Upregulated and Downregulated at day 7. | |
| --- | --- |
| Downregulated | Col4a4, Mmp15, Col4a3, Dcn, Lamb2, Jam3, Vtn, Lama2, Jam2, Nid2, Lama3, Lama5, Ddr2, Itga10, Itga7, Hspg2, Lamb1, Nid1, Lama4, Col6a3, Lum, Col6a2, Mmp2, Pdgfb, Col4a1, Sh3pxd2a, Col6a1, Col15a1, Col4a5, Mrc2, Fbln5 |
| Upregulated | Bgn, Fbn1, Col16a1, Pdgfa, Itga11, Col14a1, Sparc, Col5a1, Col8a1, Comp, Col5a2, Col12a1, Col1a2, Col3a1, Col8a2, Col1a1, Eln, Lox, Postn |
