# Supplementary material for: Mapping macrophage polarization over the myocardial infarction time continuum
Source: Basic Res Cardiol. 2018 Jun 4;113(4):26. doi: 10.1007/s00395-018-0686-x (PMC5986831; doi:10.1007/s00395-018-0686-x)
Supplement: Supplementary file 12 — Supplementary material 12 (DOCX 12 kb) [file 395_2018_686_MOESM12_ESM.docx]

| **Supplemental Table 2. Day 0 macrophage markers** | | | |
| --- | --- | --- | --- |
| **Gene** | **Average FPKM** | **Macrophage Role** | **Expression in other cell types** |
| Atf3 | 321 | Inhibition of inflammatory gene expression | ubiquitous |
| Cbr2 | 307 | M1 marker | unknown |
| Folr2 | 209 | Folate binding; M2 marker | neutrophils |
| Actr3 | 200 | LPS-induced spreading | unknown |
| Cd81 | 191 | Inhibition of motility/MMP release | ubiquitous |
|  |  |  |  |
| **Gene** | **p value** | **Macrophage Role** | **Expression in other cell types** |
| Cfh | 1.35E-08 | Complement activation; anti-inflammatory | unknown |
| Lilra5 | 6.58E-08 | Induces cytokine release | neutrophils |
| Cd209f | 2.29E-07 | M2 marker | dendritic cells |
| Cmah | 1.89E-06 | Sialic acid production; anti-inflammatory | ubiquitous |
| Tln2 | 6.08E-07 | Inhibits ECM degradation | ubiquitous |
